# Factors associated with bleeding events from enoxaparin used for patients with acute coronary syndrome

**DOI:** 10.1186/s12872-023-03278-9

**Published:** 2023-05-06

**Authors:** Adisak Weerasaksanti, Sarawut Siwamogsatham, Yotsaya Kunlamas, Krittin Bunditanukul

**Affiliations:** 1 Department of Pharmacy, King Chulalongkorn Memorial Hospital, Thai Red Cross Society, Bangkok, 10330 Thailand; 2 Clinical Research Center, Research Division, Faculty of Medicine, Chulalongkorn University, Bangkok, 10330 Thailand; 3 Department of Pharmacy Practice, Faculty of Pharmaceutical Sciences, Chulalongkorn University, Bangkok, 10330 Thailand

**Keywords:** Factors, Bleeding, Acute coronary syndrome, Enoxaparin

## Abstract

**Background:**

Low molecular weight heparins (LMWHs) are the mainstay of treatment for acute coronary syndrome (ACS). However, bleeding, the main side effect, is associated with prolonged hospitalization and mortality. Therefore, assessment of the incidence of bleeding and associated risk factors is crucial in developing an appropriate treatment plan to prevent bleeding.

**Methods:**

A retrospective cohort study was conducted in patients with ACS admitted to a university hospital in Bangkok, Thailand between 2011 and 2015 and received enoxaparin. To estimate the incidence of bleeding events, patients were followed up for 30 days from the first enoxaparin dose. Multiple logistic regression was used to determine factors associated with bleeding events.

**Results:**

From a total of 602 patients, the incidence of bleeding was 15.8%, of which 5.7% involved major bleeding. The risk factors for any form of bleeding were aged at least 65 years (odds ratio [OR], 1.99; 95% confidence interval [CI], 1.18 to 3.36), history of bleeding (OR, 3.79; 95% CI, 1.24 to 11.55), and history of oral anticoagulant exposure (OR, 4.73; 95% CI, 1.74 to 12.86).

**Conclusion:**

ACS patients treated with enoxaparin had an increased risk of bleeding if they were aged 65 years or older, had a history of bleeding events, and had a history of taking oral anticoagulants.

## Background

Acute coronary syndrome (ACS) is considered the leading cause of death in many parts of the world, including Thailand [1, 2]. The most important treatment decisions is the evaluation of ischemic and bleeding risks for optimal antithrombotic therapies and the timing of vascular reperfusion of the occluded coronary artery. Many clinical studies showed that use of parenteral anticoagulants was associated with a lower risk of adverse cardiovascular events in ACS patients [3–5]. Therefore, the American College of Cardiology (ACC)/American Heart Association (AHA) and the European Society of Cardiology (ESC) recommend parenteral anticoagulation as the standard of care for ACS [6–9].

The use of unfractionated heparin (UFH) is limited by its unpredictable effect and the need for monitoring the activated partial thromboplastin clotting time (aPTT). In contrast, low molecular weight heparins (LMWHs) e.g., enoxaparin, has a lighter structure with a longer half-life. It provides predictable anticoagulation effect without the need for monitoring. Moreover, it is less likely to cause heparin-induced thrombocytopenia [10]. In Thailand, many hospitals replace the use of UFH with enoxaparin for treatment of ACS.

Although many randomized controlled trials confirm that the use of enoxaparin for ACS could improve ischemic and bleeding outcomes [5, 11], there are some limitations. Due to the strict inclusion and exclusion criteria, the study population of clinical trials and incidence of bleeding complications were often not representative of the patients in clinical practice. To our knowledge, real-world evidence on the incidence of bleeding complications and their associated risk factors related to the use of enoxaparin for ACS in Thailand is lacking. Therefore, this study aimed to identify risk factors associated with bleeding complications from enoxaparin for the treatment of ACS in real-world clinical practice in Thailand.

## Methods

This retrospective cohort study using data from medical records of patients with ACS who received enoxaparin and were admitted to a tertiary care university hospital in Thailand between January 1, 2011 to December 31, 2015. The inclusion criteria included age at least 18 years at enrollment, diagnosed with ACS (unstable angina [UA], non-ST elevation myocardial infarction [NSTEMI], or ST-elevation myocardial infarction [STEMI]) and received at least one dose of enoxaparin. Exclusion criteria were receipt of thrombolytic or glycoprotein IIb/IIIa inhibitor, use of intravenous infusion UFH after starting enoxaparin, and those who did not have 30-day follow-up data after the first dose of enoxaparin.

### Variable definition

Dose of enoxaparin was collected as dose in milligram (mg) per actual weight (kg). The daily dose of enoxaparin in this study was adjusted according to the recommended initial daily dose of 1 mg/kg every 12 h for patients with a creatinine clearance (CrCl) ≥ 30 mL/min and 1 mg/kg every 24 h for patients with a CrCl < 30 mL/min [9]. CrCl was estimated using Cockcroft-Gault formula (mL/min). Use of non-steroidal anti-inflammatory (NSAIDs) drugs was defined as history of NSAIDs exposure in one month before receiving the first dose of enoxaparin. Use of oral anticoagulant (OAC) was defined as OAC use of at least seven days with the last dose within 24 h prior to switching to enoxaparin. History of bleeding was defined as having any bleeding events (medication or non-medication related) in one year prior to initiation of enoxaparin. Anemia was defined as hemoglobin (Hb) less than 13 g/dL in male and 12 g/dL in female. Heart failure was ascertained by signs and symptoms at admission. Doses of enoxaparin were categorized into three subgroups: recommended dose (0.9 to 1.1 mg/kg/dose), lower than recommended dose (< 0.9 mg/kg/dose), and higher than recommended dose (> 1.1 mg/kg/dose) based on renal function and body weight [12].

### Outcomes

The primary outcomes were incidence of bleeding and factors associated with bleeding from enoxaparin. Bleeding events were classified by appearance or site and assessed for their severity using Bleeding Academic Research Consortium (BARC type 1 to 5). Bleeding events which fulfilled BARC criteria type 3 to 5 would be considered major bleeding. This study followed patients for 30 days after initiation of enoxaparin. Patients who were bleeding during this follow-up period were classified into two groups: bleeding during treatment and bleeding after discontinuation of the drug. If multiple bleeding episodes occurred simultaneously, only the most severe episode will be recorded. The secondary outcome was enoxaparin dosing at the authors’ institute.

### Potential risk factors for bleeding

A preliminary list of patient and treatment characteristics were identified as potential determinants of bleeding risk: age ≥ 65 years, SBP > 160 mmHg, CrCl (mL/min), anemia (male < 13 g/dL, female < 12 g/dL), cerebrovascular disease, history of bleeding, history of NSAIDs use, history of OAC use, number of enoxaparin doses, types of P2Y_12_ receptor inhibitors received, procedure for coronary intervention and the area where the coronary artery catheter was inserted, and other underlying diseases.

The variables above were obtained from three sources including (1) medical records on the present admission (recorded by a physician), (2) medication reconciliation form which all patients were asked about history of medication before admission by a pharmacist, doctor, or nurse, and (3) medical records from other hospitals.

If there were no data found in these sources, the researcher assumes that patients did not have those variables.

### Statistical analysis

Baseline characteristics were reported as frequency and percent for categorical variables, while mean (SD) or median (IQR) were reported for continuous variables. Differences in baseline characteristics between patients with bleeding events and those without were compared using X^2^ test for categorical variables and independent t-test or Man-Whitney-U test for continuous variables. Sample size was estimated by event per variable rule: *N* = 10 k/p, k was the number of variables [i.e. age ≥ 65 years; SBP > 160 mmHg; CrCl (mL/min); Anemia; cerebrovascular disease; history of bleeding; history of NSAIDs use; history of oral anticoagulant use; number of enoxaparin doses; types of P2Y_12_ receptor inhibitors received; procedure for coronary intervention and the area where the coronary artery catheter was inserted] and p was the probability of bleeding in ACS patients receiving enoxaparin which was about 0.183 [13]. The k and p in this study was 11 and 0.183, respectively. Thus, the estimated sample size was 602 patients. The odds ratio (OR) and corresponding 95% confidence interval (CI) were reported for each variable. Variables with *p*-value < 0.1 were included in the multivariable model. Multivariate logistic regression with forward selection likelihood ratio (LR) was used for adding variables in the model (*p* < 0.05). All analyses were performed using SPSS software version 20. Data collection was cross checked by two other cardiology pharmacists in the hospital. We used Listwise deletion method for handling of missing data.

## Results

### Baseline characteristics

**Fig. 1:**
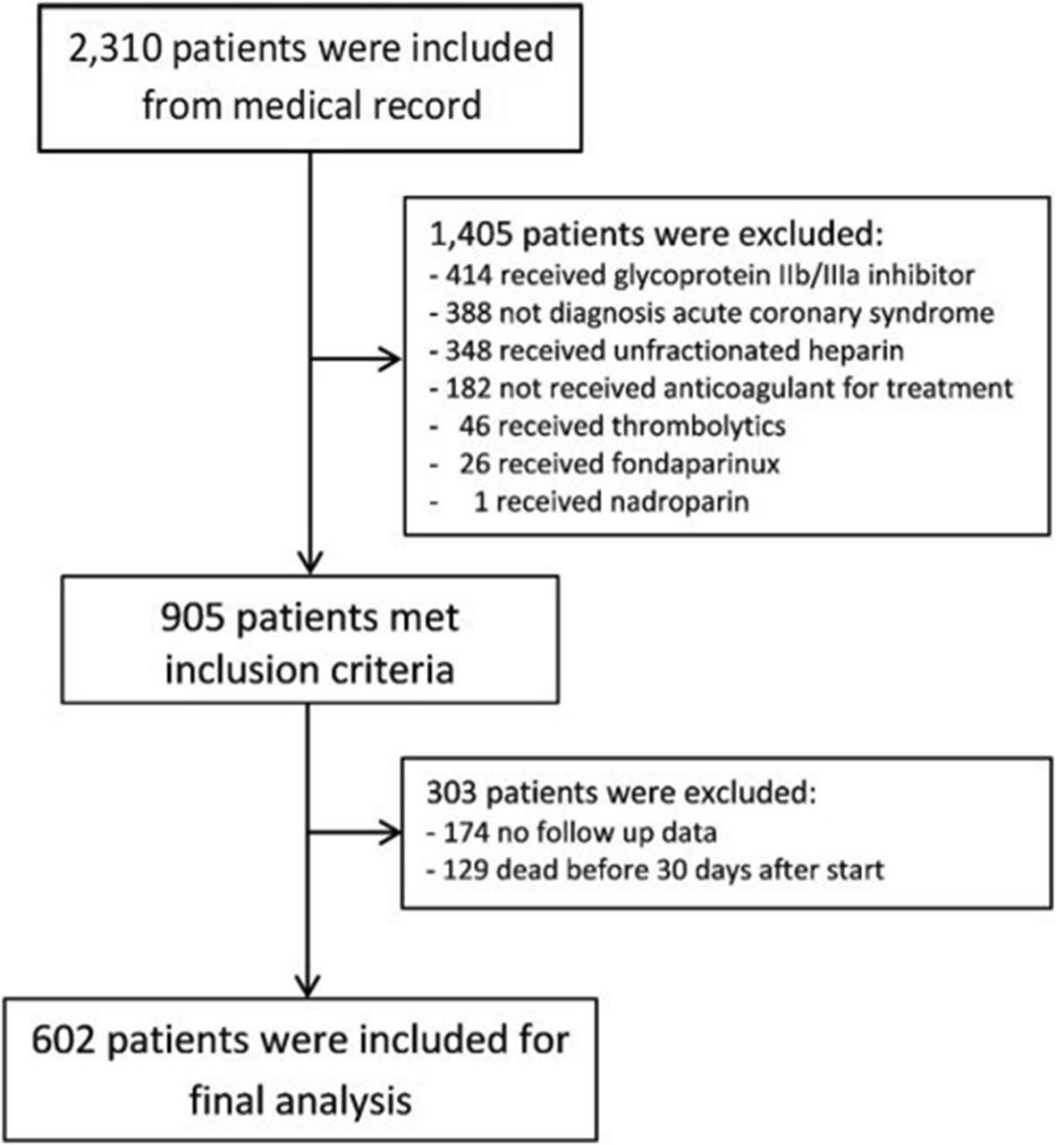
Flow chart for patient inclusion and exclusion

A total of 602 ACS patients were included in this study for data analysis (Fig. 1). Of those, 3.3%, 70.1%, and 26.6% had UA, NSTEMI, and STEMI, respectively. A comparison of baseline demographics and laboratory characteristics between bleeding and non-bleeding groups are presented in Table 1. Overall, the mean age was 68.2 years (SD ± 12.0), 65.3% of the patients were at least 65 years old, and about 63% were males. One hundred and one patients (16.8%) had CrCl below 30 mL/min. Seventeen patients (2.8%) had a history of oral OAC exposure (all patients were receiving warfarin) and nineteen patients (3.2%) had a history of NSAIDs exposure. Coronary angiography (CAG), percutaneous coronary intervention (PCI), and coronary artery bypass graft (CABG) were performed in 78.9%, 62.5%, and 9.6% of patients, respectively. All patients received aspirin 300 mg loading dose then 81 mg once daily. Clopidogrel, ticagrelor, and prasugrel were prescribed in 90.4%, 6.6%, and 0.3% of the cases. The mean duration of enoxaparin therapy was 4.3 days, the median number of doses was six doses and the mean dose was 0.91 mg/kg. Overall, approximately 50% of the patients received enoxaparin with the prescribed dose lower than the recommended dose (< 0.9 mg/kg). Enoxaparin dosing practice is presented in Table 2.

**Table 1:** Baseline characteristics

	All patients (*n* = 602)	Bleeding Events		*p*-value
		Bleeding (*n* = 95)	Non-bleeding (*n* = 507)	
Male sex, n (%)	381 (63.3)	56 (59.0)	325 (64.1)	0.339
Age, years (mean ± SD)	68.2 ± 12.0	70.4 ± 9.7	67.8 ± 12.4	0.025
Age ≥ 65 years, n (%)	393 (65.3)	74 (77.9)	319 (62.9)	0.005
Weight, kg (mean ± SD)	62.9 ± 12.9	61.9 ± 11.5	63.0 ± 13.2	0.441
BMI, kg/m^2^ (mean ± SD)	24.1 ± 4.0	23.8 ± 3.6	24.2 ± 4.1	0.340
SBP, mmHg (mean ± SD)	136.5 ± 27.2	135.7 ± 31.2	136.6 ± 26.4	0.786
SBP ≥ 160 mmHg, n (%)	130 (21.6)	25 (26.3)	105 (20.7)	0.223
Heart rate, bpm (mean ± SD)	83.8 ± 18.6	84.6 ± 18.3	83.6 ± 19.1	0.638
CrCl, mL/min (mean ± SD)	54.6 ± 26.0	52.8 ± 23.5	55.0 ± 26.4	0.453
CrCl < 30 mL/min, n (%)	101 (16.8)	16 (16.8)	85 (16.8)	0.985
Hemoglobin, g/dL (mean ± SD)	12.6 ± 4.3	12.2 ± 2.4	12.8 ± 4.6	0.200
Platelet, × 10^3^ cells/µL (mean ± SD)	253.7 ± 89.0	245.5 ± 95.4	255.2 ± 87.8	0.328
WBC, × 10^3^ cells/ µL (mean ± SD)	10.3 ± 4.2	10.2 ± 4.0	10.3 ± 4.3	0.882
Medical history, n (%)				
Hypertension	395 (65.6)	72 (75.8)	323 (63.7)	0.023
Diabetes mellitus	286 (47.5)	47 (49.5)	239 (47.1)	0.676
Coronary artery disease	221 (36.7)	41 (43.2)	180 (35.5)	0.155
Cerebrovascular disease	91 (15.1)	20 (21.1)	71 (14.0)	0.078
History of bleeding in 1 year	14 (2.3)	6 (6.3)	8 (1.6)	0.014
History of oral anticoagulant used	17 (2.8)	8 (8.4)	9 (1.8)	0.002
History of NSAIDs used	19 (3.2)	7 (7.4)	12 (2.4)	0.019
Signs and symptoms at presentation, n (%)				
Anemia	299 (49.7)	50 (52.6)	249 (49.1)	0.529
Signs of heart failure	244 (40.5)	37 (39.0)	207 (40.8)	0.732
Diagnosis of ACS, n (%)				
Unstable angina	20 (3.3)	4 (4.2)	16 (3.2)	0.539
NSTEMI	422 (70.1)	69 (72.6)	353 (69.6)	0.557
STEMI	160 (26.6)	22 (23.2)	138 (27.2)	0.411
In-hospital management, n (%)				
Conservative strategy	91 (15.1)	21 (22.1)	70 (13.8)	0.038
CAG	475 (78.9)	68 (71.6)	407 (80.3)	0.057
Femoral access	439 (92.4)	65 (95.6)	374 (91.9)	0.286
Radial access	36 (7.6)	3 (4.4)	33 (8.1)	Ref
PCI	297 (49.3)	47 (49.5)	250 (49.3)	0.977
CABG	59 (9.8)	8 (8.4)	51 (10.1)	0.622
P2Y_12_ receptor inhibitor, n (%)				
Clopidogrel	544 (90.4)	81 (85.3)	463 (91.3)	0.066
Prasugrel	2 (0.3)	0 (0)	2 (0.4)	1.000
Ticagrelor	40 (6.6)	9 (9.5)	31 (6.1)	0.228

*Abbreviations*: *SD* Standard deviation, *BMI* Body mass index, *SBP* Systolic blood pressure, *CrCl* Creatinine clearance, *WBC* White blood cell, *NSAIDs* Non-steroidal anti-inflammatory drugs, *ACS* Acute coronary syndrome, *NSTEMI* Non-ST elevation myocardial infarction, *STEMI* ST-elevation myocardial infarction, *CAG* Coronary artery angiography, *Ref* Reference category, *PCI* Percutaneous coronary intervention, *CABG* Coronary artery bypass graft

**Table 2:** Enoxaparin dosing practice

	All patients (*n* = 602)	Bleeding Events		*p-*value
		Bleeding (*n* = 95)	Non-bleeding (*n* = 507)	
Dose, mg/kg (mean ± SD)	0.91 ± 0.25	0.94 ± 0.24	0.91 ± 0.25	0.302
Dose of enoxaparin, n (%)				
Recommended dose	255 (42.4)	43 (16.9)	212 (83.1)	0.532
Lower than recommended dose	292 (48.5)	39 (13.4)	253 (86.6)	0.113
Excess than recommended dose	55 (9.1)	13 (23.6)	42 (76.4)	0.094
Total dose, mg/kg (mean ± SD)	6.83 ± 7.32	6.24 ± 6.48	6.94 ± 7.47	0.393
Duration of enoxaparin therapy, days (mean ± SD)	4.3 ± 4.4	3.7 ± 3.6	4.4 ± 4.5	0.151
Doses administered, doses (IQR)	6 (3–9)	5 (3–8)	6 (3–9)	0.238

*Abbreviations*: *SD* Standard deviation, *IQR* Interquartile range

### Incidence and characteristics of bleeding events

There were 95 bleeding events (15.8%), of which 34 (5.7%) involved major bleeding (BARC 3a to 5b). The most common bleeding characteristic was hematoma, followed by hematuria and hemoptysis or bleeding per endotracheal tube. Two fatal bleeding events included retroperitoneal and pericardial bleeding. The incidence and characteristics of bleeding events are presented in Table 3. Eighty-three bleeding episodes (87.4%) were observed when patients received enoxaparin and the median duration of bleeding was three days after starting the medication. Twelve bleeding events (12.6%) occurred after discontinuation of enoxaparin, of which, 10 bleeding events developed within 24 to 48 h after discontinuation of enoxaparin. There was no difference in the incidence of bleeding in patients who received recommended enoxaparin dose compared to those who received the lower than recommended dose (16.9% and 13.4%; OR 1.31, 95% CI: 0.82 to 2.11) or higher than the recommended dose (16.9% and 23.6%; OR 0.66, 95% CI: 0.32 to 1.32). Likewise, we did not find any differences in the risk of bleeding between those in improper dose and recommended dose groups.

**Table 3:** Incidence and characteristics of bleeding events

	No. (%)
Severity of bleeding	
Any bleeding	95 (15.8)
Minor bleeding (BARC 1 to 2)	61 (10.1)
Major bleeding (BARC 3a to 5b)	34 (5.7)
Bleeding characteristics	
Hematoma	41 (43.2)
Hematuria	15 (15.8)
Hemoptysis or bleeding per endotracheal tube	10 (10.5)
Post coronary artery bypass graft bleeding	5 (5.3)
Upper gastrointestinal bleeding	5 (5.3)
Intracranial bleeding	3 (3.2)
Melena	3 (3.2)
Pericardial effusion	2 (2.0)
Retroperitoneal bleeding	2 (2.0)
Lower gastrointestinal bleeding	2 (2.0)
Groin bleeding	2 (2.0)
Pleural effusion	1 (1.1)
Percutaneous transhepatic biliary drainage bleeding	1 (1.1)
Bleeding per gum	1 (1.1)
Epistaxis	1 (1.1)
Bleeding per colostomy	1 (1.1)

### Risk factors for bleeding

Risk factors of bleeding, based on the univariable analysis, are presented in Table 4. Nine factors associated with bleeding (*p* < 0.1) were identified. However, the correlation coefficient of the procedure received in ACS treatment and CAG factor was 0.793 and was removed from the model. The remaining eight factors were included in the analysis. In the multivariable analysis (forward selection likelihood ratio), risk factors of any bleeding (*p* < 0.05) are presented in Table 5. Three risk factors that were independently associated with any bleeding events were age ≥ 65 years, history of bleeding within one year, and history of oral anticoagulant exposure.

**Table 4:** Risk factors for any bleeding (univariable analysis)

Characteristics	Odds ratio (95% CI)	*p-*value
Age ≥ 65 years	2.08 (1.24–3.48)	0.005*
SBP > 160 mmHg	1.37 (0.83–2.27)	0.223
Creatinine clearance, mL/min	1.00 (0.99–1.01)	0.453
Cerebrovascular disease	1.64 (0.94–2.85)	0.078*
Hypertension	1.78 (1.08–2.95)	0.023*
History of bleeding	4.21 (1.43–12.41)	0.014*
History of NSAIDs used	3.28 (1.26–8.56)	0.019*
History of oral anticoagulant used	5.09 (1.91–13.55)	0.002*
Anemia (Hemoglobin: male < 13 g/dL, female < 12 g/dL)	1.15 (0.74–1.79)	0.529
Conservative strategy	1.77 (1.03–3.06)	0.038*
Coronary artery angiography (CAG)	0.62 (0.38–1.02)	0.057*
Femoral access^a^	1.91 (0.57–6.42)	0.286
Percutaneous coronary intervention (PCI)	1.01 (0.65–1.56)	0.977
Clopidogrel	0.55 (0.29–1.05)	0.066*
Ticagrelor	1.61 (0.74–3.49)	0.228
No. of enoxaparin doses	0.98 (0.95–1.02)	0.282

^a^Reference group = radial access

^*^p-value < 0.1

**Table 5:** Risk factors for any bleeding (multivariable analysis, forward selection likelihood ratio)

Characteristics	Adjusted odds ratio (95% CI)	*p-*value
Age ≥ 65 years	1.99 (1.18–3.36)	0.010
History of bleeding	3.79 (1.24–11.55)	0.019
History of oral anticoagulant used	4.73 (1.74–12.86)	0.002

## Discussion

In this retrospective cohort study, we assessed real-world incidence and factors associated with bleeding events from enoxaparin use in ACS patients. Bleeding can prolong hospitalization and even be fatal. No previous studies have reported on this issue based on current data in Thailand. Therefore, identifying factors associated with bleeding occurrence will be useful for developing an appropriate treatment plan to prevent bleeding. In this study, the mean age of patients was 68 years old and most of them were diagnosed with NSTEMI. The population profile was similar to those in the large randomized-controlled trials of enoxaparin for the treatment of ACS [4, 14–16] and previous retrospective studies in Thai patients [13]. The most common underlying diseases were hypertension, diabetes mellitus, and coronary heart disease; all of which are risk factors of ACS. Patients in the bleeding group were older and had a larger proportion of patients ≥ 65 years old. In addition, more patients in the bleeding group had a history of hypertension, history of bleeding, history of anticoagulant use, history of NSAIDs use, and conservative strategy for ACS treatment than the non-bleeding group. In this study, 48.5% of patients received a lower than recommended dose of enoxaparin. This is higher than the patients in another study of the same focus, which only reported 15%. However, the proportion of patients receiving higher than recommended doses was similar to those two studies (8% and 11%) [12]. The mean dose of enoxaparin in this study was 0.91 mg/kg, which was similar between groups.

In this study, nearly all patients were treated with P2Y_12_ receptor inhibitors and almost all were clopidogrel. Due to cost limitations and public health policy in Thailand at the time of the study, access to other P2Y_12_ receptor inhibitors was limited. CAG was performed in approximately 80% of the cases and almost all of them had a catheter in the groin area. There were only a few patients who had the catheter inserted at the radial access site. This could be due to the suitability of the patients and the physician's aptitude for the procedure.

 The incidence of bleeding in our study was 15.8%. This is similar to the incidence reported in a study by Macie and colleagues [12]. The incidence of bleeding events in two large randomized controlled trials, the Efficacy and Safety of Subcutaneous Enoxaparin in Non–Q-wave Coronary Events study group (ESSENCE) and the Thrombolysis In Myocardial Infarction (TIMI)11B, were 18.4% and 22%, respectively [4, 14]. These were slightly higher than what was reported in our study. In addition, the incidence of major bleeding in our study was about 5.7%, which was similar to a previous study (4%) [12] and a meta-analysis of enoxaparin in ACS patients (4.7%) [17]. However, another retrospective study in Thailand [13] reported the incidence of major bleeding to be 18.3%, which was higher. This is likely due to the inclusion of patients who received thrombolytic and glycoprotein IIb/IIIa inhibitor. The most common bleeding characteristic was hematoma (43.2%), followed by hematuria (15.8%) and hemoptysis or bleeding per endotracheal tube (10.5%). Two fatal bleeding events, pericardial and retroperitoneal bleeding, occurred while the patients were treated with enoxaparin.

Several risk factors related to bleeding were identified in the current study population. First, patients ≥ 65 years old (65.3% of the study patients) had two folds increased risk of bleeding (adjusted OR, 1.99; 95% CI, 1.18 to 3.36). This is consistent with the previous study which reported the increased risk of bleeding with increasing age (OR, 1.57; 95% CI, 1.13 to 2.20) [12].

Second, history of OAC use was the strongest predictor of bleeding events in our study (adjusted OR, 4.73; 95% CI, 1.74 to 12.86). In a previous study, no relationship between OAC use and bleeding events was found [12]. One possible reason may be because of a small sample size. In that study, only about 30% of patients used P2Y_12_ inhibitor [12] while almost all patients in our study received P2Y_12_ inhibitor. Furthermore, the mean of the INR measurements within 24 h before initiation of enoxaparin was recorded and evaluated. The INR values of participants who had history of warfarin use between the bleeding and non-bleeding groups in our study were not significantly different (2.22 ± 1.03 and 2.12 ± 1.06; *p* = 0.863). However, we did not evaluate INR after enoxaparin initiation. Some patients with ACS may also go into a shock which may affect liver function and metabolism of warfarin to enhance its anticoagulant effect.

The last factor that was related to bleeding was history of bleeding. This increased the risk of bleeding by 3.79 times (95% CI, 1.24 to 11.55). Patients with a history of bleeding are considered a high-risk group because they may have ulcers, lesions, or tissue vulnerability that can lead to re-bleeding. The previous study did not include this factor in the analysis [12].

The factors that was found to be associated with bleeding in the previous study [12] but not in this study was the number of exposures to enoxaparin. In this study, the cumulative dose of enoxaparin was also analyzed, which found that neither factor was associated with bleeding. In real-life practice, patient’s body weight can be underestimated or overestimated and consequently lead to ordering enoxaparin at a lower or higher dose than recommended. This was reported in a previous study in Thailand [13]. Therefore, the incidence of bleeding might also be underestimated. The non-difference in the incidence of bleeding between patients who received high dose and normal dose of enoxaparin may be due to the small sample size in the high-dose group than in the recommended dose group, relatively short duration of enoxaparin exposure, and other factors predisposed to bleeding.

Theoretically, type of P2Y_12_ inhibitor could be associated with bleeding but evidence on this factor is limited. In 2004, guidelines for the treatment of acute myocardial ischemia did not recommend P2Y_12_ receptor inhibitors for all ACS patients [12], but the current treatment guidelines suggest that this should be prescribed to all patients. In this study, 90% of the patients were treated with clopidogrel. According to the national drug list and universal coverage insurance of Thailand, most of our ACS patients received clopidogrel in combination with aspirin except in STEMI patients treated with primary PCI, GRACE risk score > 140 for NSTEMI patients, or patients who were allergic to clopidogrel. These patients would receive ticagrelor instead of clopidogrel. The use of high potent P2Y_12_ inhibitor may be associated with a higher risk of bleeding than our study. This gap can lead to further studies. However, factors associated with bleeding from enoxaparin in this study could still be considered for ACS patients who received enoxaparin independent of the type of P2Y_12_ inhibitors in their treatment regimen. Type of P2Y_12_ inhibitor and the number of exposures to enoxaparin were included in the univariate analysis with the risk of bleeding; however, we did not find different risk of bleeding from both factors which may be due to small population. Because previous studies have shown that those factors might affect bleeding, they should be considered when evaluating the risk of bleeding from enoxaparin and also need further studies. The insertion site of coronary artery catheterization can likely influence different bleeding risks. However, almost all patients in this study were performed in the groin, this factor may not play a role.

The risk factors identified in this study differed from all CRUSADE scores [18], and these factors should be included in the assessment of bleeding. The findings from this study may be used to determine the appropriate protocol or regimen of enoxaparin therapy in high-risk bleeding ACS patients and to select appropriate interventions that reduce the risk of bleeding. Moreover, some institutions may use our results to help develop a protocol for bleeding precautions such as close monitoring of bleeding events, monitoring hemoglobin/hematocrit levels, or schedule discharge from the hospital for at least 24 to 48 h after receiving the last dose of enoxaparin in patients who have these risk factors.

## Limitations

Several limitations of our study should be acknowledged. First, this study was a retrospective study. It was not possible to correct or adjust treatment, when patients received an inappropriate dose of enoxaparin. Therefore, if the patient’s weight was not accurately estimated, this could impact the study outcomes. In our study, approximately 50% of patients received enoxaparin with lower than the recommended dose, this could result in a lower incidence of bleeding. Second, missing data, a common problem for retrospective studies, may affect the results of the study. In our study there was less than 5% of missing data and the researchers also used Listwise deletion method to handle those missing data. Third, this study was a single-center study, so the results may not be generalizable to other population where treatment patterns and patient characteristics are different. Fourth, this study's goal was only to assess safety but did not monitor efficacy. Such issues are equally important in planning the appropriate treatment of patients. Finally, this study collected data from 2011 to 2015. Some of the current treatment guidelines may be different, such as type of P2Y_12_ receptor inhibitor use, duration of dual antiplatelet therapy, as well as CAG or PCI proficiency and expertise that may affect outcomes. Therefore, further studies are needed.

## Conclusions

In this study, the incidence of enoxaparin-associated bleeding in Thai ACS patients is 15.8%. Patient’s age of ≥ 65 years, history of bleeding events, and history of oral anticoagulants were major determinants of bleeding. Therefore, enoxaparin use should be closely monitored in patients with one of these bleeding determinants.

## Data Availability

The datasets are available from the corresponding author on reasonable request.

## Abbreviations

ACS: Acute coronary syndrome
aPTT: Activated partial thromboplastin clotting time
BARC: Bleeding Academic Research Consortium
BMI: Body mass index
CABG: Coronary artery bypass graft
CAG: Coronary angiography
CI: Confidence interval
CrCl: Creatinine clearance
Hb: Hemoglobin
IQR: Interquartile range
LMWHs: Low molecular weight heparins
LR: Likelihood ratio
NSTEMI: Non-ST elevation myocardial infarction
NSAIDs: Non-steroidal anti-inflammatory drugs
OR: Odds ratio
OAC: Oral anticoagulant
PCI: Percutaneous coronary intervention
SBP: Systolic blood pressure
SD: Standard deviation
STEMI: ST elevation myocardial infarction
UFH: Unfractionated heparin
UA: Unstable angina
WBC: White blood cell
